# A 50% higher prevalence of life-shortening chronic conditions among cancer patients with low socioeconomic status

**DOI:** 10.1038/sj.bjc.6605949

**Published:** 2010-10-26

**Authors:** W J Louwman, M J Aarts, S Houterman, F J van Lenthe, J W W Coebergh, M L G Janssen-Heijnen

**Affiliations:** 1 Comprehensive Cancer Centre South (IKZ), Eindhoven Cancer Registry, PO Box 231, Eindhoven 5600 AE, The Netherlands; 2 Department of Public Health, Erasmus University Medical Center Rotterdam, PO Box 2040, Rotterdam 3000 CA, The Netherlands; 3 MMC Academy, Máxima Medical Center, PO Box 7777, Veldhoven 5500 MB, The Netherlands

**Keywords:** socioeconomic status, comorbidity, chronic disease, survival

## Abstract

**Background:**

Comorbidity and socioeconomic status (SES) may be related among cancer patients.

**Method:**

Population-based cancer registry study among 72 153 patients diagnosed during 1997–2006.

**Results:**

Low SES patients had 50% higher risk of serious comorbidity than those with high SES. Prevalence was increased for each cancer site. Low SES cancer patients had significantly higher risk of also having cardiovascular disease, chronic obstructive pulmonary diseases, diabetes mellitus, cerebrovascular disease, tuberculosis, dementia, and gastrointestinal disease. One-year survival was significantly worse in lowest *vs* highest SES, partly explained by comorbidity.

**Conclusion:**

This illustrates the enormous heterogeneity of cancer patients and stresses the need for optimal treatment of cancer patients with a variety of concomitant chronic conditions.

People of a lower socioeconomic status (SES) generally have poorer health status and higher mortality than people of higher SES (Jemal *et al*, 2008; Mackenbach *et al*, 2008), also with respect to cancer, with in general higher incidence rate of all cancers combined among people from lower socioeconomic groups (Dalton *et al*, 2008). A differential distribution of known risk factors for specific neoplasms between SES groups seems a likely explanation for the above inequalities. For example, the prevalence of smokers has become higher among lower classes (Lahelma *et al*, 1997; Stronks *et al*, 1997), probably resulting in higher rates of cancer of the lung, larynx, mouth, pharynx, oesophagus, and bladder (Siemiatycki *et al*, 1995; Stellman and Resnicow, 1997; Tyczynski *et al*, 2003). However, smoking is not only related to cancer but also to chronic obstructive pulmonary diseases (COPD) and cardiovascular diseases (Doll *et al*, 1994). Hence, the high prevalence of comorbidity among lung cancer patients (Janssen-Heijnen *et al*, 1998). Socioeconomic status may thus be associated with comorbidity among cancer patients. Thus, medical doctors are presented with a heterogeneous group of cancer patients, for whom appropriate individual treatment must be chosen, taking concomitant conditions into account (Ayanian *et al*, 2003; Lash *et al*, 2003; Janssen-Heijnen *et al*, 2004; Lemmens *et al*, 2005; Louwman *et al*, 2005; van Spronsen *et al*, 2005).

We studied in a large population-based group of cancer patients the prevalence of comorbidity according to SES, not only by number of concomitant diseases, but also for specific diseases that affect patients with the various tumour sites.

## Materials and methods

The Eindhoven Cancer Registry records data on all patients newly diagnosed with cancer in the south of the Netherlands (2.4 million inhabitants, 15% of the Dutch population); it also records serious comorbidity according to an adaptated list (Charlson *et al*, 1987). Chronic obstructive pulmonary diseases, cardio- and cerebrovascular diseases, peripheral arterial disease, other malignancies, and diabetes mellitus, connective tissue diseases, rheumatoid arthritis, kidney, bowel, and liver diseases, dementia, tuberculosis and other chronic infections were also recorded. For most analyses peripheral arterial disease was included in the cardiovascular diseases, although gastrointestinal diseases were grouped (gastric diseases, Crohn's disease, ulcerative colitis, liver cirrhosis, and hepatitis). Comorbidity was defined as life-shortening disease that was present at the time of cancer diagnosis and/or received treatment or surveillance. Trained registry personnel actively collect data on diagnosis, staging, and treatment from the medical records after notification by pathologists and medical registration offices. Previous admissions, letters from and to general practitioners and other specialists, the medical history and preoperative screening were used as sources.

Patients with cancer of the oesophagus, stomach, colon or rectum, pancreas, lung, melanoma, breast, cervix uteri, corpus uteri, ovary, prostate, bladder, kidney, and non-Hodgkin's lymphoma (NHL), newly diagnosed between 1997 and 2006 (*n*=72 153), were included in this study; cancers diagnosed at autopsy (*n*=369) were excluded.

Statistics Netherlands developed an indicator of SES, using individual fiscal data on the economic value of the home and household income, and is provided at aggregated level for each postal code (covering an average of 17 households). Socioeconomic status was categorised as low (deciles 1–3), medium (deciles 4–7), or high social class (deciles 8–10), and a separate class for postal codes for a long-term care providing institution (such as a nursing home; van Duijn and Keij, 2002). We calculated the distribution of cancer patients across socioeconomic strata according to tumour localisation, also by gender and age. Patients for whom the SES was unknown (*n*=766, 1%) or for whom the postal code included a care providing institution (*n*=3569, 5%), as well as those with unknown comorbidity (*n*=8399, 12%) were excluded from the analyses of SES and comorbidity. Differences in distribution were tested with the *χ*^*2*^ test. Logistic regression analyses of the odds of having a specific concomitant disease were performed age- and gender-adjusted for all tumour sites combined, and according to tumour site for four concomitant diseases separately; cardiovascular disease, COPD, diabetes mellitus, and gastrointestinal disease. Statistical significance of an overall effect of SES on the prevalence of a specific condition was tested using the *χ*^2^-likelihood ratio test. Crude 1-year survival rates were calculated for all studied tumours combined and for the most important tumour sites separately. Cox's regression models were used to compute multivariate rates (hazard ratio=HR) and 95% confidence intervals (95% CI). The relative contribution (%) of adding comorbidity to the model was calculated as follows: ((HR model A−HR model B)/(HR model A−1)) × 100, where A is the basic model (age- and gender-adjusted) and in model B comorbidity is added to model A. All statistical analyses were performed using SAS V9.12 (SAS Institute Inc., Cary, NC, USA).

## Results

Male cancer patients were older than female patients (Table 1), the median age being 69 and 64 years, respectively (*P*<0.0001). At the time of the diagnosis of the cancer 71% of male and 58% of female cancer patients had at least one concomitant disease. The most frequent concomitant condition for males with cancer was cardiovascular disease (23%), for women hypertension (20%), among cancer patients older than 70 the prevalence of these diseases was 34% and 31%, respectively. In the subgroup of cancer patients with two or more concomitant diseases, the most frequent combination of diseases among males was cardiovascular disease with hypertension (14%) and in females diabetes with hypertension (21%).

The proportion of patients by SES varied for the different tumour sites (Table 2). Patients under age 70 with stomach, lung, bladder, or cervical cancer more often had low SES. High SES was more frequent among patients with melanoma or breast, colorectal, or prostate cancer in this age group.

Among patients aged 70+ with cancer of the oesophagus, stomach, or lung, low SES was clearly over-represented. High SES was more frequent among patients with prostate cancer or NHL.

**Figure 1:**
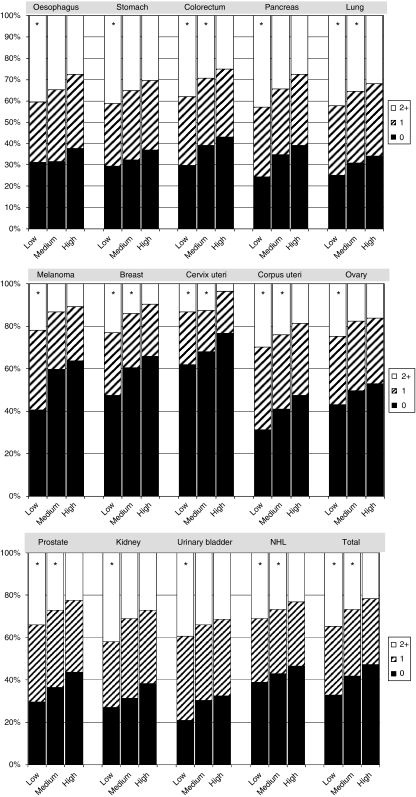
Number of concomitant diseases among cancer patients diagnosed in 1997–2006 in the Southeastern Netherlands. ^*^Distribution of number of concomitant diseases significantly different from the highest socioeconomic status category.

For all tumour localisations the proportion of patients without comorbidity was highest in the high SES group (Figure 1). A gradient towards more concomitant conditions appeared in lower SES groups (*P*<0.001), which had a significantly higher risk of cardiovascular disease (OR_low *vs* high SES_=1.4, 95% CI: 1.3–1.5), COPD (OR=1.8 (1.7–1.9)), diabetes mellitus (OR=1.5 (1.4–1.6)), cerebrovascular disease (OR=1.5 (1.4–1.7)), tuberculosis (OR=1.3 (1.1–1.6)), dementia (OR=1.3 (1.0–1.8)), gastrointestinal disease (OR=1.5 (1.4–1.6)), and two or more concomitant conditions (OR=1.8 (1.7–1.9)) in addition to their cancer (Table 3). The risk of having cancer and also at least one other serious concomitant disease was 50% higher in the low SES than in the high SES group (OR=1.5 (1.4–1.6)).

**Figure 2:**
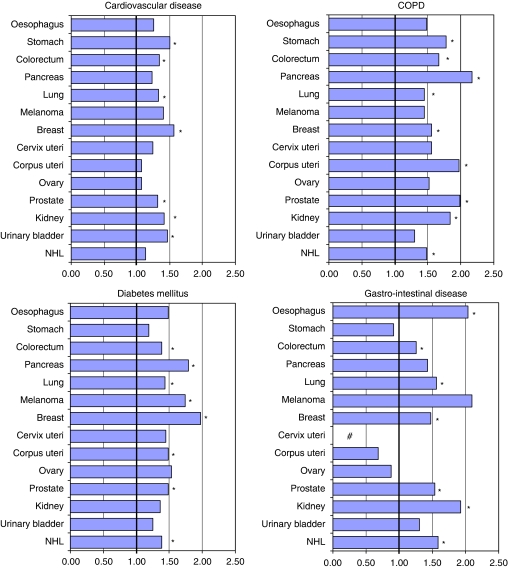
Risk of four concomitant diseases among cancer patients with the lowest socioeconomic status (SES) compared with those with the highest SES (=reference, 1.00) according to tumour localisation with adjustment for age and gender. ^*^95% confidence interval does not include 1.00; # No reliable estimate because <5 cases in reference category.

For four concomitant conditions we stratified by tumour localisation (Figure 2). The risk of cardiovascular disease among low compared with high SES patients was significantly higher (1.4–1.6 times) for patients with stomach, colorectal, lung, breast, prostate, and bladder cancer. The risk of COPD was elevated among low SES patients with cancer of the stomach, colorectum, pancreas, lung, breast, corpus uteri, prostate, and kidney (OR's ranging from 1.4 to 2.2). The risk of diabetes mellitus was highest among people from low SES with breast cancer (OR=2.0 (1.2–2.4)) and the risk of gastrointestinal diseases was highest among patients with oesophageal cancer (OR=2.0 (1.2–3.4)).

Crude 1-year survival of cancer patients from lower SES was worse compared with the highest SES for all tumour sites combined and for the major sites separately (Table 4). The age-adjusted risk of death was significantly elevated for both men (HR_low *vs* high SES_=1.40, 95% CI: 1.3–1.4) and women (HR 1.40 (1.3–1.5)). Adding comorbidity to the model reduced HR to 1.35 for men and 1.34 for women. The relative contribution of comorbidity in explaining the inequality in 1-year survival varied from 0% for lung cancer to 33% among female colorectal cancer patients.

## Discussion

To our knowledge, this is the first large population-based study to demonstrates the impact of SES on the prevalence of concomitant diseases among cancer patients, with increased prevalence of comorbidity in lower socioeconomic strata for each type of cancer. Cancer patients with low SES had a 50% higher risk of suffering from at least one other serious disease compared with those with high SES. The prevalence of comorbidity was significantly higher with newly diagnosed cancer of lower compared with higher SES for all 14 cancer sites studied. The diseases significantly related to SES among cancer patients were cardiovascular disease, COPD, diabetes mellitus, cerebrovascular disease, tuberculosis, diseases of the central nervous system, and gastrointestinal disease. Although both the prevalence of comorbidity and the proportional distribution of SES vary significantly among tumour types, the gradient of more comorbidity from high to low SES was apparent among all tumour types.

Smoking is probably responsible for the higher risk of cardiovascular disease, COPD, and cerebrovascular disease among low SES groups (Doll *et al*, 1994; Stellman and Resnicow, 1997). This is confirmed by the higher prevalence of those diseases among patients with smoking-related tumours: cancers of the stomach, lung, bladder, and kidney (Janssen-Heijnen *et al*, 1998; Koppert *et al*, 2004). Diabetes was more frequent among low SES for patients with cancers of the colorectum, pancreas, lung, breast, corpus uteri or prostate, or melanoma or NHL. Diabetes has been linked to pancreas cancer (Jain *et al*, 1991; Kalapothaki *et al*, 1993) either as a risk factor or as the clinical manifestation of the cancer itself (Warshaw and Fernandez-del Castillo, 1992). Diabetes has also been associated with an increased risk for breast (Xue and Michels, 2007), endometrial (Parazzini *et al*, 1991), and colorectal cancer (Polednak, 2006) probably because of a relation with obesity (Reeves *et al*, 2007). Substantial evidence exists for the association of obesity with low SES (Sobal and Stunkard, 1989; Wardle *et al*, 2002; McLaren, 2007).

The prevalence of gastrointestinal diseases was highest for low SES patients with oesophageal, colorectal, lung, breast, prostate or kidney cancer, or NHL. Oesophageal cancer has also been associated with gastrointestinal diseases (Koppert *et al*, 2004). A lower consumption of vegetables, fruit, and fibres, which may protect from oesophageal (Tzonou *et al*, 1996; Terry *et al*, 2001b) and colorectal cancer (Pietinen *et al*, 1999; Michels *et al*, 2000; Voorrips *et al*, 2000; Terry *et al*, 2001a; Bueno-de-Mesquita *et al*, 2002; Flood *et al*, 2002), has been reported among lower SES (Wallstrom *et al*, 2000; Hulshof *et al*, 2003; Wardle and Steptoe, 2003).

We used an indicator of SES based on the postal code of a residential area. This aggregate covers a very small geographical area, and thus represents a reliable approximation of individual SES. Furthermore, routinely collected income tax data (no questionnaires or interviews) have been found to provide reliable estimates of household income. Previous studies have proven that socioeconomic differences based on neighbourhood data tend to reflect such differences well at the individual level (Bos *et al*, 2000, 2001; Smits *et al*, 2001). Furthermore, this objective measure of SES is also applicable to older women (born before 1955), whose occupation or education does not always properly reflect their social class (Berkman and Macintyre, 1997).

Previously, we found that patients with comorbidity were often treated less aggressively, if alternative treatment strategies were available. Except for patients with a tumour with poor survival, comorbidity has an independent prognostic effect (Janssen-Heijnen *et al*, 2005). This negative impact of comorbidity on survival of cancer might have several mechanisms: the increased risk of death due to the comorbid condition itself, more contra-indications for the cancer treatment, more indications for dose reduction and a higher rate of treatment-related complications such as infections and cardiovascular events. In several of our recent studies, the adverse effects of comorbidity on survival appeared to be independent of treatment, so less aggressive treatment could not (fully) account for the observed differences in survival between patients with and without comorbidity (Post *et al*, 2001; Lemmens *et al*, 2005; Louwman *et al*, 2005; van Spronsen *et al*, 2005; Houterman *et al*, 2006). As SES represents a combination of lifestyle, health, and risk of suboptimal treatment, cancer patients with comorbidity could also (partly) explain the poorer prognosis. Although an in-depth study remains necessary to reveal whether stage at diagnosis and treatment contributed to the SES gradient in survival, also for longer survival periods, our preliminary analyses demonstrated a clear gradient in 1-year survival rates, which could partly be attributed to comorbidity.

Our study shows considerable variation in comorbidity by tumour type and a higher risk of concomitant disease among patients from lower SES. Given the aetiology of the type of tumours as well as the aetiology of the concomitant diseases that occur more frequently among patients from low SES background, a lot can probably be gained from preventive measures related to lifestyle (such as smoking and obesity). Considering survival is worse for patients of low SES, our results stress the need for reduction of socioeconomic differences in health.

## Figures and Tables

**Table 1:** Description of all cancer patients diagnosed with selected tumours between 1997 and 2006 in the Eindhoven Cancer Registry

	**Males**		**Females**		**Total**	
	***n***	**%**	***n***	**%**	***n***	**%**
*Tumour localisation*						
Oesophagus	1079	3	398	1	1477	2
Stomach	1723	5	1032	3	2755	4
Colorectal	6815	19	6014	17	12 829	18
Pancreas	907	2	849	2	1756	2
Lung	9354	26	3591	10	12 945	18
Melanoma	1405	4	1899	5	3304	5
Breast	—		14 859	41	14 859	20
Cervix uteri	—		725	2	725	1
Corpus uteri	—		2128	6	2128	3
Ovary	—		1540	4	1540	2
Prostate	9987	27	—		9987	14
Kidney	1201	3	806	2	2007	3
Urinary bladder	2306	6	679	2	2985	4
Non-Hodgkin's lymphoma	1846	5	1413	4	3259	4
						
*Age*						
<45	1156	3	3884	11	5040	7
45–59	6624	18	10 578	29	17 202	24
60–74	18 984	52	13 142	37	32 126	44
>75	9859	27	8329	23	18 188	25
						
*SES*						
Low	9518	26	9953	28	19 471	27
Intermediate	14 309	39	13 824	38	28 133	39
High	10 812	30	9741	27	20 553	28
Institution	1569	4	2032	6	3601	5
Unknown	415	1	383	1	798	1
						
*Comorbidity*						
*Number of concomitant diseases*						
0	10 688	29	14 826	41	25 514	35
1	10 775	29	9353	26	20 128	28
>2	10 992	30	7050	20	18 042	25
Unknown	4168	11	4704	13	8872	12
						
*Concomitant disease*^a^						
Previous cancer	4460	12	3565	10	7977	11
Cardiovascular disease	8353	23	3854	11	12127	17
Peripheral arterial disease	3445	9	1358	4	4767	7
COPD	5347	15	2674	7	7994	11
Hypertension	6367	17	7184	20	13 462	19
Diabetes mellitus	3586	10	3482	10	7026	10
Cerebrovascular disease	1754	5	1044	3	2779	4
Tuberculosis	553	2	409	1	947	1
Central nervous system^b^	221	1	354	1	568	1
Gastrointestinal disease	2629	7	1294	4	3900	5
Other diseases	925	3	1078	3	1987	3
Total	36 623	50	35 933	50	72 556	100

Abbreviations: COPD=chronic obstructive pulmonary diseases; SES=socioeconomic status.

a Patients may suffer from more than one condition.

b Dementia in 96% of these patients.

**Table 2:** Distribution of cancer patients newly diagnosed in 1997–2006 according to gender, age and socioeconomic status (SES)

	**Males**				**Females**			
	**<70**		**70+**		**<70**		**70+**	
**Tumour localisation**	**No. of patients**	**% low SES**	**No. of patients**	**% low SES**	**No. of patients**	**% low SES**	**No. of patients**	**% low SES**
Oesophagus	589	23	342	37	170	30	161	43
Stomach	767	26	719	37	386	31	465	44
Colorectal	3176	21	2662	32	2266	24	2630	40
Pancreas	433	24	339	36	325	28	374	44
Lung	4498	29	3827	38	2226	35	923	50
Melanoma	563	15	169	33	729	16	194	43
Breast	—	—	—	—	9070	21	3094	42
Cervix uteri	—	—	—	—	476	37	120	43
Corpus uteri	—	—	—	—	1189	25	576	42
Ovary	—	—	—	—	875	23	438	42
Prostate	3930	20	4149	30	—	—	—	—
Kidney	639	22	392	32	372	31	300	41
Urinary bladder	855	25	1027	33	216	32	308	40
Non-Hodgkin's lymphoma	1060	21	575	31	703	25	514	42
Total of these sites	16 510	23	14 201	34	19 003	24	10 097	42

**Table 3:** Risk of specific concomitant diseases according to SES adjusted for age and gender among cancer patients diagnosed in 1997–2006

	**SES**			
**Concomitant disease**	**Low**	**Intermediate**	**High**	***P* a**
Previous cancer	1.01	0.99	1.00	0.7
Cardiovascular disease	1.42^b^	1.23^b^	1.00	0.0001
COPD	1.81^b^	1.37^b^	1.00	0.0001
Hypertension	0.98	1.03	1.00	0.2
Diabetes mellitus	1.52^b^	1.32^b^	1.00	0.0001
Cerebrovascular disease	1.53^b^	1.27^b^	1.00	0.0001
Tuberculosis	1.34^b^	1.17	1.00	0.01
Central nervous system	1.34^b^	1.05	1.00	0.05
Gastrointestinal	1.48^b^	1.27^b^	1.00	0.0001
Other	1.22^b^	1.10	1.00	0.01
1 or more concomitant disease	1.50^b^	1.24^b^	1.00	0.0001
2 or more concomitant diseases	1.80^b^	1.36^b^	1.00	0.0001

Abbreviations: COPD=chronic obstructive pulmonary diseases; SES=socioeconomic status.

a *P* for overall effect of SES (χ^2^-likelihood ratio).

b Confidence interval does not include 1.00.

**Table 4:** Crude survival, risk of death, and contribution of comorbidity to risk of death according to tumour site and SES among cancer patients diagnosed in 1997–2006

	**1-year survival rate (%)**			**Model A^a^**	**Model B^a^**	**Relative contribution**
	**Low SES**	**Inter-mediate**	**High SES**	**HR^c^ (95% CI)**	**HR^c^ (95% CI)**	**comorbidity^b^**
*Males*						
Colorectum	72	78	78	1.13 (1.0–1.3)	1.10 (1.0–1.3)	23%
Lung	36	39	41	1.11 (1.0–1.2)	1.11 (1.0–1.2)	0%
Prostate	90	94	95	1.47 (1.2–1.8)	1.36 (1.1–1.7)	22%
Total^d^	59	66	70	1.40 (1.3–1.5)	1.35 (1.3–1.4)	12%
						
*Females*						
Colorectum	74	78	79	1.09 (0.9–1.3)	1.06 (0.9–1.2)	33%
Lung	41	42	46	1.09 (1.0–1.2)	1.09 (1.0–1.2)	0%
Breast	94	97	98	1.68 (1.3–2.2)	1.56 (1.2–2.0)	18%
Total^d^	74	81	84	1.40 (1.3–1.5)	1.34 (1.3–1.4)	15%

a Model A: adjusted for age, Model B: adjusted for age and the presence of concomitant diseases (yes *vs* no).

b ((HR model A−(HR model A+comorbidity))/(1−HR model A)) × 100.

c Hazard Ratio (HR) of lowest socioeconomic status (SES) group compared with highest (=reference).

d All studied sites combined (oesophagus, stomach, colorectum, pancreas, lung, melanoma, breast, cervix uteri, corpus uteri, ovary, prostate, kidney, urinary bladder, non-Hodgkin's lymphoma).
